# Navigating Value Co-Destruction in Open Innovation Communities: An Empirical Study of Expectancy Disconfirmation and Psychological Contracts in Business Analytics Communities

**DOI:** 10.3390/bs13040334

**Published:** 2023-04-17

**Authors:** Mohammad Daradkeh

**Affiliations:** 1 College of Engineering and Information Technology, University of Dubai, Dubai 14143, United Arab Emirates; mdaradkehc@ud.ac.ae or mdaradkeh@yu.edu.jo; 2 Faculty of Information Technology and Computer Science, Yarmouk University, Irbid 21163, Jordan

**Keywords:** open innovation communities, expectancy disconfirmation theory, value co-creation, value co-destruction, user-enterprise interaction, psychological contract breach, business analytics

## Abstract

Enterprises seeking to enhance their innovation capabilities are increasingly turning to open innovation communities (OICs), which allow them to leverage the collective knowledge and collaborative potential of external users, providing a powerful source of new and innovative ideas. Despite their potential for value co-creation, recent research suggests that value co-destruction can also occur within OICs. However, the mechanisms underlying value co-destruction in OICs have not yet been fully explored or empirically examined. To address this gap, this study employs expectancy disconfirmation theory and psychological contract theory to investigate the relationship between user expectancy disconfirmation and value co-destruction in OICs. Drawing upon data collected from a questionnaire survey of business analytics OICs, this study reveals that self-interest expectancy disconfirmation has a positive effect on value co-destruction, which is mediated by the transactional psychological contract breach. In addition, social interaction expectancy disconfirmation is found to have a positive impact on value co-destruction, which is mediated by the relational psychological contract breach. The study further reveals that self-worth expectancy disconfirmation of community users positively influences value co-destruction, which is mediated by the ideological psychological contract breach. Moreover, the study demonstrates the crucial role of perceived organizational status in moderating the ideological psychological contract breach resulting from self-worth expectancy disconfirmation. Collectively, these findings contribute valuable insights into the phenomenon of value co-destruction in OICs, and provide practical guidance for enterprises seeking to enhance the development and performance of these innovation paradigms.

## 1. Introduction

The landscape of innovation is in a constant state of flux, demanding that businesses keep pace with the rapid advancements in technology and expedite their product development cycles. However, this presents a challenge for enterprises that solely rely on their internal resources and innovation capabilities. In response to this, open innovation has emerged as a leading strategy that enables businesses to leverage external resources, enhance their knowledge base and creative outputs, and remain competitive [1]. One of the most powerful means for tapping into the vast collective knowledge and collaborative potential of external users is through open innovation communities (OICs). These OICs are recognized as a critical ingredient for success in the world of enterprise innovation as users provide valuable feedback for product enhancement, solutions for product flaws, and preferences for new products, including novel ideas that can be incorporated into the enterprise’s products, making them the most extensive source of innovation. As such, many companies have implemented different types of OICs to incorporate external knowledge and contributions at different stages of innovation development, such as ideas, patents, products, and business models [2]. These contributions are ones that the company may never have created due to a lack of time, internal knowledge, and resources. Among these different types of OICs, firm-hosted OICs, such as Microsoft Power BI Community, Tableau Community, Huawei Community, GitHub, OpenIDEO, and Mozilla, have gained increasing popularity. The basis for decentralized cooperation among members is formed through the openness of knowledge and the exclusion of proprietary exploitation patterns, characterizing the form of coordination of innovation communities. Moreover, these communities are directly focused on the products and services of the individual companies, and members contribute by suggesting innovative ideas or simply by sharing their preferences and needs [3,4,5].

Since open innovation communities (OICs) are characterized by openness and cooperation, the issue of value co-destruction, which refers to the situation where the inappropriate use of resources leads to a reduction in one party’s welfare [1], is inherent to these communities. While OICs has been viewed as a means for firms to leverage external resources, expand their knowledge base, and maintain competitiveness, recent research indicates that interactions between enterprises and users may result in value co-destruction rather than value co-creation [6,7]. Consequently, it has become imperative for businesses to explore the mechanisms underlying this phenomenon to optimize the benefits of open innovation while minimizing potential risks. Studies have shown that inconsistencies in practice elements may lead to value co-destruction for both enterprises and consumers, and project managers’ lack of attention to customers and misalignment of values between them can lead to value co-destruction. Numerous studies have delved into the intricacies of value co-destruction in digital communities. For instance, Arici et al. [8] discovered that collective service experiences within leisure industry communities can be misappropriated, demonstrating the ubiquity of value co-optation in online communities. In addition, Bidar et al. [9] established that both consumer experience failures and corporate misconduct can trigger value destruction in online communities. Ashraf et al. [10] also identified user feedback provision and help-seeking behaviors as potential sources of value co-destruction in online communities. Adequate communication and congruence among interacting parties, as pointed out by Chang et al. [11], has been recognized as another significant factor contributing to value co-destruction. In the realm of OICs, users typically participate with the expectation of contributing their knowledge and innovative ideas in exchange for tangible or intangible rewards. However, there may be a perceptual gap below the expected reference point due to disparities between their expectations and the enterprise’s practice elements, which leads to the perception that the enterprise has failed to fulfill its obligations. This breach of the psychological contract can lead to the cessation of knowledge and creative contributions, resulting in value co-destruction [4,12,13,14].

Although there has been a growing interest in exploring the mechanisms of value co-destruction in open innovation communities (OICs), research in this area is still in its nascent stage [15]. To advance our understanding of value co-destruction in OICs, it is imperative to address several key research gaps and challenges, including:

- Limited empirical research: Despite growing interest in exploring the mechanisms of value co-destruction in OICs, there is a scarcity of empirical research in this area. Existing studies primarily rely on qualitative research methods, with few large-scale empirical studies conducted to fully comprehend the complex mechanisms of value co-destruction within OICs.
- Inadequate understanding of online communities: The majority of research on value co-destruction has focused on offline contexts, resulting in a significant gap in our understanding of value co-destruction within online communities. Given the increasing prevalence of OICs in today’s digital landscape, it is imperative to gain a deeper understanding of the unique factors that contribute to value co-destruction in these contexts.
- Limited focus on user-business interactions: Current research has predominantly been conducted from a marketing perspective that emphasizes the interactions between businesses and consumers, overlooking the crucial role of users as external employees. Businesses actively motivate user participation and deliver innovation value by treating users as external employees within OICs, highlighting the need for a more comprehensive understanding of the user-business interactions within OICs.
- Absence of organizational behavior and user expectancies perspective: Despite the fact that businesses function as external operators and managers within OICs, seeking to motivate user participation in innovation and deliver innovation value, there has been a lack of attention paid to the organizational behavior and community user expectancies perspective. Incorporating this perspective will provide a more complete understanding of the role of businesses as external operators within OICs and how they can optimize the benefits of value co-creation while avoiding the problem of value co-destruction.

This study proposes a novel theoretical model that seeks to address gaps in current research by examining the mechanisms underlying enterprise-user value co-destruction in OICs. It is a theoretical model that employs expectancy disconfirmation theory [16] and psychological contract theory [17,18] as lenses for analysis is proposed, treating value co-destruction as an independent variable. Furthermore, the model integrates psychological contract breach as a mediating variable and organizational status perception as a moderating variable. The study employs a quantitative research approach, using a survey method to gather data from users of four business analytics OICs. The analysis of the gathered data, using multiple regression analysis on a sample of 321 community users, reveals that self-interest expectancy disconfirmation positively influences value co-destruction through the mediation of transactional psychological contract breach, while social interaction expectancy disconfirmation positively impacts value co-destruction through the mediation of relational psychological contract breach. Furthermore, the study discovers that self-worth expectancy disconfirmation of community users positively influences value co-destruction, mediated by the ideological psychological contract breach. Finally, the study emphasizes the pivotal role of perceived organizational status in moderating the ideological psychological contract breach resulting from self-worth expectancy disconfirmation.

This study contributes to the field of innovation management by elucidating the mechanisms of value co-destruction in OICs, which have emerged as a popular platform for enterprise-user collaborations. Through the provision of a theoretical model and empirical evidence, this study offers insights to enterprises seeking to optimize the benefits of value co-creation and mitigate the negative consequences of value co-destruction. The key contributions of this study are summarized as follows:

- The study proposes a novel theoretical framework that integrates expectancy disconfirmation theory, providing a comprehensive understanding of the intrinsic mechanisms that govern enterprise-user value co-destruction in OICs. The proposed model, which links expectancy disconfirmation, psychological contract breach, and value co-destruction in OICs, represents a significant contribution to the literature.
- The study enhances the theoretical and practical understanding of the mediating and moderating roles played by psychological contract breach and organizational status perception in enterprise-user value co-destruction in OICs. This valuable insight can help organizations identify factors contributing to value co-destruction and develop effective strategies to prevent its occurrence.
- Lastly, the study offers practical guidance to organizations grappling with the recurrent challenge of value co-destruction in OICs. Through the understanding and management of enterprise-user value co-destruction in OICs, organizations can surmount obstacles to innovation and improve their growth performance.

The rest of this paper is structured as follows. Section 2 provides a comprehensive theoretical foundation for the study by exploring topics such as value co-creation and co-destruction, expectancy disconfirmation, psychological contract breach, and perceived organizational status. In addition, this section presents the research hypotheses. Section 3 outlines the research methodology, which includes sample selection, data collection and analysis methods, and instrument measures. In Section 4, the results of the data analysis are presented, including testing for common method variance, instrument validity and reliability, correlation analysis, and hypothesis testing. Section 6 discusses the implications of the results for both the field and practical applications of the study. Section 7 identifies the study’s limitations and proposes future research directions. Finally, the paper concludes in Section 8 by summarizing the main findings and their significance.

## 2. Theoretical Foundation and Research Hypotheses

### 2.1. Value Co-Destruction in OICs

Since the emergence of service-dominant logic, which emphasizes that firms do not create value but rather claim it, there has been a shift towards a more comprehensive understanding of value creation that recognizes customers as active value co-creators [19]. However, not all interactions between firms and their customers lead to positive outcomes, and research has identified value co-destruction as a phenomenon that is more likely to occur than value co-creation [20]. Value co-destruction, first introduced by Plé et al. [19], refers to the situation where the interaction between two parties (service systems) results in a reduction in the welfare of at least one party due to the inappropriate use of resources. Based on the original definition proposed by Plé et al. [19], this study conceptualizes value co-destruction in OICs as the process of enterprise–user interaction in which users perceive that the enterprise has failed to appropriately and efficiently integrate or utilize resources from both sides, resulting in a decline in their willingness to share knowledge and, ultimately, leading to a reduction in innovation sources and outcomes for the enterprise. The multifaceted path to value co-destruction in OICs calls for the development of effective strategies to prevent its occurrence [21,22].

The literature has identified several factors contributing to value co-destruction in OICs, including misaligned expectations, power imbalances, and ineffective resource management. Managing value co-destruction in OICs requires distinctive strategies, such as the development of collaborative governance frameworks, the cultivation of shared norms and values, and the utilization of mediation and conflict resolution techniques [1,2]. Enterprises seek to attract and engage users to obtain sources and outcomes of innovation, but value co-destruction may arise when users perceive a failure to integrate or utilize resources from both sides [23,24]. Codá et al. [25] examined the types of online resources that are available to both users and enterprises in open innovation communities (OICs), and the impact that these resources have on the growth and performance of contribution in such communities. The authors identified two types of online resources that are available to users: informational resources and social resources. Informational resources refer to the knowledge, information, and expertise that users possess and share with the community. Social resources, on the other hand, refer to the social capital that users have, such as the relationships, networks, and trust that they build with other members of the community. The authors found that the availability and utilization of these online resources had a significant impact on the growth and performance of contribution in OICs.

Qualitative research on the causes and processes of value co-destruction has been conducted based on the practice theory-informed research, along with individual case studies. For instance, Dolan et al. [26] demonstrate that inconsistencies in practice elements may lead to value co-destruction for both enterprises hosting OICs and online users. Echeverri et al. [27] suggests that a lack of attention to contributors by managers of OICs and misalignment of values between them can also lead to value co-destruction. Engen et al. [22] provide a detailed observation and dynamic description of the value co-destruction process between operators of OICs and community members based on the approach/avoidance motivation of members. Frau et al. [28] argue that improper OIC marketing and inflated customer expectations can also result in mutual value co-destruction. These studies highlight the diverse nature of value co-destruction and underscore the need for further research to understand the factors triggering its occurrence and underlying mechanisms of the process.

### 2.2. Expectancy Disconfirmation Theory (EDT)

Expectancy Disconfirmation Theory (EDT) is a widely studied theoretical framework that has been applied to various fields, including marketing, consumer behavior, and organizational behavior. The inception of EDT can be attributed to the pioneering work of Oliver [29], who proposed the theory in the context of consumer behavior. Oliver [29] argued that consumer satisfaction is dependent on the discrepancy between the expected and actual performance of a product or service. He posited that individuals create expectations based on their prior experiences, advertising, and word-of-mouth communication, which serve as a reference point for their subsequent evaluations of a product or service. The EDT suggests that individuals create expectations based on a reference point and compare their actual perceived performance with this reference point [16]. If the perceived performance falls short of the expected reference point, individuals may experience negative disconfirmation and dissatisfaction. This can lead to reluctance to engage in innovation activities and value co-destruction, which can have adverse consequences for the enterprise.

According to the EDT, managing user expectations and aligning them with the enterprise’s innovation activities is critical for value co-creation and long-term success. Enterprises need to recognize that user expectations are formed through individualized comparative judgments based on a reference frame, and that negative disconfirmation can occur when perceived performance falls short of the expected reference point. Therefore, it is important for enterprises to communicate clearly with users about their expectations for innovation activities, and to ensure that these expectations are realistic and achievable. To achieve this, enterprises should engage in ongoing dialogue with their users, and seek to understand their expectations and motivations for participating in innovation activities. This may involve conducting user surveys or focus groups, or engaging in one-on-one discussions with key users. By doing so, enterprises can gain a deeper understanding of the types of rewards and incentives that are most important to their users, and can develop strategies for aligning these incentives with the enterprise’s innovation goals.

In addition, enterprises should also be transparent in their communication with users, and should provide regular feedback on their performance in innovation activities. This can help to manage user expectations by setting realistic goals and benchmarks for performance, and by ensuring that users are aware of the progress they are making towards these goals. Regular feedback can also help to build trust and credibility with users, and can help to foster a sense of community and collaboration among users. In essence, individuals create expectations by comparing what they receive (i.e., actual perceived performance) with what they create (i.e., expected reference point). When perceived performance falls below the desired reference point, individuals experience negative disconfirmation [30,31]. EDT has been utilized to examine the interplay between enterprise and user expectations and their influence on value co-destruction. EDT contends that expectations are shaped based on a reference point, which functions as the standard for evaluating perceived performance. Enterprises can establish enduring, collaborative relationships with their users and foster a sustainable culture of innovation and creativity by aligning user expectations with enterprise innovation activities [16].

### 2.3. Expectancy Disconfirmation and Value Co-Destruction in OICs

In the context of OICs, managing expectations is critical to prevent value co-destruction. By understanding the reference points of users in OICs, enterprises can take proactive measures to meet their expectations and avoid negative disconfirmation. For example, a collaborative governance framework that fosters shared norms and values may be established to facilitate interactions between the enterprise and users. Additionally, mediation and conflict resolution methods can be employed to address any conflicts or misunderstandings that arise between the enterprise and users. Kandampully et al. [32] argue that users of online OICs expect to receive returns in the form of economic and social benefits. Similarly, this study asserts that users of OICs have expectations for both economic and social benefits, specifically, the desire to gain self-interest expectancy disconfirmation benefits (such as money, points, vouchers, etc.) and satisfy their social interaction needs (for example, participating in group activities, meeting new people, etc.) through knowledge sharing within the community [31,33,34].

Furthermore, users in OICs exhibit unique characteristics, with some displaying a high level of enthusiasm for corporate mission construction activities [30] and expecting no rewards in return [35]. As a result, users in OICs possess expectations that transcend profit and social interaction, specifically, the expectation to contribute to the enterprise’s noble mission or greater vision, which Liang [36] refer to as self-worth expectancy. For example, members of a healthcare community in the healthcare industry may envision that their contributions will assist the enterprise in fulfilling its mission and vision of advancing humanity’s welfare [37]. Thus, enterprises need to take into account the diverse expectations of OIC users to effectively engage with them and create value together while avoiding co-destruction. Given the diverse expectations of users in OICs, including economic, social, and self-value expectations, failure to manage expectations effectively can lead to value co-destruction, thereby jeopardizing enterprise-user collaborations [38]. To this end, enterprises seeking to leverage OICs must gain a comprehensive understanding of the reference points that underpin users’ expectations to proactively provide tailored solutions that align with users’ expectations and forestall negative disconfirmation [39].

The present study posits that a gap between user expectations and community innovation activities can have adverse consequences for value creation in OICs. Specifically, users who hold self-interest expectations anticipate receiving extrinsic rewards such as financial compensation, points, and honorary titles for their knowledge sharing efforts [40]. However, the enterprise may fail to deliver on the promised rewards or distribute virtual rewards that lack tangible value, leading to a misalignment between user expectations and actual rewards received, and resulting in hesitation to dedicate time and effort towards innovation activities they perceive as “futile” [41].

Similarly, users who hold social interaction expectations anticipate receiving social support, friendship, and intimate relationships through their knowledge sharing efforts [42]. However, the enterprise’s innovation tasks may not incorporate collaborative elements or the lack of interest in the innovation activities, leading to a misalignment between user social interaction expectations and actual experience, and resulting in reluctance to engage in innovation activities they perceive as “lonely” [43].

Finally, users who hold self-worth expectations anticipate contributing to the company’s advocated values and lofty missions through their knowledge sharing efforts [44]. However, due to a disconnect between the enterprise’s innovation activities and mission building or the lack of enthusiasm in participating in mission-building activities, leading to a misalignment between user self-value expectations and actual experience, and resulting in unwillingness to invest time and effort in innovation activities they perceive as “mediocre” [45].

Based on the above, three hypotheses are posited in the present study:

**H1a.**  *There exists a positive correlation between self-interest expectancy disconfirmation and value co-destruction. The greater the discrepancy between self-interest expectations and the actual rewards received, the higher the likelihood of value co-destruction.*

**H1b.**  *There exists a positive correlation between social interaction expectancy disconfirmation and value co-destruction. The greater the discrepancy between social interaction expectations and the actual experience of the activities, the higher the likelihood of value co-destruction.*

**H1c.**  *There exists a positive correlation between self-worth expectancy disconfirmation and value co-destruction. The greater the discrepancy between self-worth expectations and the actual experience of the activities, the higher the likelihood of value co-destruction.*

### 2.4. The Mediating Role of Psychological Contract Breach

In the realm of organizational behavior, the psychological contract theory is a seminal concept that captures the beliefs and expectations of employees regarding their mutual obligations with their employers. This theory can be classified into two primary schools of thought: the classical school [18,46] and the Rousseau [17]. The former describes the psychological contract as a mutual understanding of obligations between both parties, while the latter emphasizes that the psychological contract is an understanding and belief of unilateral obligations among employees [47,48].

In the context of OICs, the psychological contract is a critical component that shapes the relationships between community users and the community enterprise. Adopting the Rousseau school’s perspective, the psychological contract is perceived as the commitment and belief of community users towards their own contributions and the exchange of returns with the community enterprise. However, unlike traditional employment relationships, the interaction between the enterprise and users in an OIC is not based on a written contract but rather on a psychological contract that is often fragile due to the lack of contractual constraints and legal protection [49].

In such scenarios, the breach of the psychological contract occurs when users perceive that the enterprise has not fulfilled its obligations as expected [50]. In addition to the widely recognized transactional and relational dimensions, this study suggests that an ideological dimension also exists in the research context [19,51]. This dimension is based on the belief of some users in the pursuit of lofty social goals, which implicitly exists in the reciprocity between individuals and the organization. This implies that users believe that the community enterprise has the responsibility to strive for noble mission ideals or at least to provide a working environment that enables users to contribute to the realization of these ideals [52,53].

When users perceive that the enterprise has not fulfilled its promises, it leads to a breach of the psychological contract. Specifically, when users with a transactional psychological contract perceive that the benefits which they receive are smaller than expected, they will conclude that they have fulfilled their obligation of contributing knowledge to the community, but the enterprise has not rewarded them in a way that matches their contributions. When users with a relational psychological contract feel that their social interaction experience is lower than expected, they will think that the enterprise does not value or has not fulfilled its obligation to create a friendly community atmosphere. When users with an ideological psychological contract feel that their self-worth realization is lower than expected, they will think that the enterprise has not fulfilled its promise to pursue a mission [51,54].

These breaches of psychological contract can lead to negative outcomes, such as value co-destruction, which refers to the phenomenon in which the value created by users is destroyed by the enterprise [18]. Therefore, the study proposes the following hypotheses:

**H2a.**  *Transactional psychological contract breach mediates the relationship between self-interest expectancy disconfirmation and value co-destruction.*

**H2b.**  *Relational psychological contract breach mediates the relationship between social interaction expectancy disconfirmation and value co-destruction.*

**H2c.**  *Ideological psychological contract breach mediates the relationship between self-worth expectancy disconfirmation and value co-destruction.*

### 2.5. The Moderating Effect of Perceived Organizational Status

Perceived organizational status, as defined in the field of organizational behavior research, refers to an individual’s self-evaluation of their value and status within an organization [48]. This perception significantly affects the attitudes and behaviors of employees [55]. Studies indicate that employees with a high perceived organizational status tend to be more committed to their organization and feel a greater sense of responsibility to contribute towards achieving its goals and mission [56]. In the context of OICs, the concept of perceived organizational status is particularly relevant as it can influence community members’ interactions and contributions towards the community’s goals and mission. When community members perceive themselves as high-status members, they are more likely to experience a sense of ownership and responsibility towards the community, leading to increased commitment and engagement with the community [56]. They may also assume leadership roles, becoming advocates for the community’s interests and taking a more proactive approach to addressing any issues or opportunities for improvement. Additionally, these individuals may engage in constructive communication with other community members and stakeholders, fostering a collaborative and positive environment for innovation and problem-solving [12].

However, in the event of expectancy disconfirmation experienced by online users, community members with high perceived organizational status may feel more qualified and obligated to communicate with community-led enterprises to fulfill their obligations and mitigate undesirable community conditions. In the case of self-interest expectancy disconfirmation, community members with high perceived organizational status tend to believe that they possess the necessary resources and qualifications for innovation activities and may persuade the community to increase reward levels to prevent transactional psychological contract breaches. Similarly, in the case of social interaction expectancy disconfirmation, community members with high perceived organizational status tend to have a deeper understanding of the community atmosphere, more representative wishes, and may act as representatives for the community to advise enterprises and prevent relational psychological contract breaches. Finally, in the case of self-worth expectancy disconfirmation, community members with high perceived organizational status may consider themselves responsible for leading community members to achieve the community’s mission and may adopt strong advising behaviors to prevent ideological psychological contract breach. In summary, perceived organizational status plays a crucial role in OICs, affecting community members’ attitudes and behaviors towards their community [27,31]. This study aims to explore how perceived organizational status moderates the relationships between different types of expectancy disconfirmation and psychological contract breaches in OICs. Therefore, this study proposes three hypotheses:

**H3a.**  *Perceived organizational status moderates the relationship between self-interest expectancy disconfirmation and transactional psychological contract breach, with a weaker positive relationship between the two as perceived organizational status increases.*

**H3b.**  *Perceived organizational status moderates the relationship between social interaction expectancy disconfirmation and relational psychological contract breaches, with a weaker positive relationship between the two as perceived organizational status increases.*

**H3c.**  *Perceived organizational status moderates the relationship between self-worth expectancy disconfirmation and ideological psychological contract breach, with a weaker positive relationship between the two as perceived organizational status increases.*

**Figure 1:**
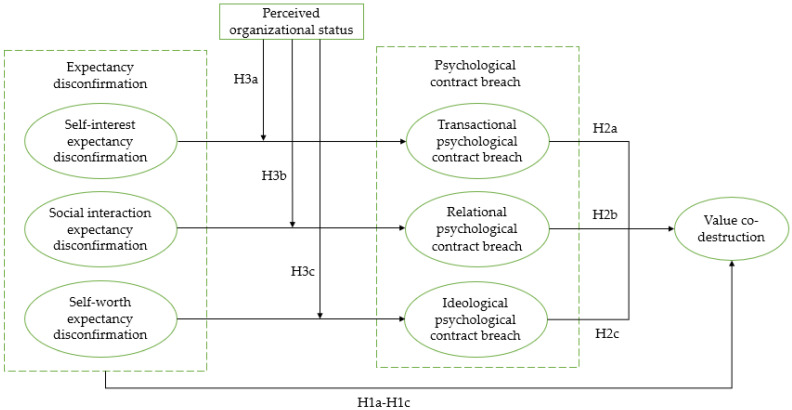
Research Model.

The model of this study is depicted in Figure 1.

## 3. Research Methodology

### 3.1. Sampling and Data Collection

This study collected research data from four open innovation communities (OICs) in business analytics, including the Microsoft Power BI Community, Tableau Community, KNIME Community, and Qlik Community. These communities were selected for this study based on the following criteria. First, the company’s innovation performance data is publicly available. For example, as of January 2023, the Microsoft Power BI Community has successfully collected 9241 ideas, of which 938 have been successfully implemented, representing 10% of the total number of ideas submitted. Second, these OICs has a large amount of published content, user comments and other interactive data. Third, these communities have a large and diverse user base, with a growing number of users searching, contributing, and exchanging knowledge within the community every day. For example, as of January 2023, the Microsoft Power BI Community has more than 50 million registered users, with more than 10,000 users active every day [2,3,57]. Therefore, it is convenient to find a sufficient sample of contributors to study and compare the interaction dynamics between the motivating factors of value co-destruction in the OIC. In addition, the large user base provided a valuable source of data for previous academic studies [4,5,58]. Therefore, as successful professional OICs, these innovation communities can serve as a typical case study for academic research and provide useful suggestions for the development of other OICs [4].

The analysis of these OICs reveals several key characteristics of their social structures. First, the user engagement and participation in these communities are high, with a large number of users regularly contributing and exchanging knowledge. This high level of user engagement is fostered by the community managers, who provide various incentives and recognition programs to encourage users to participate and contribute to the community. Second, the social structures of these OICs are characterized by a high degree of openness and transparency. Users are encouraged to share their ideas and feedback openly, and community managers are responsive to user suggestions and concerns. This openness and transparency foster a culture of trust and collaboration, which in turn enhances the value co-destruction process within the community. Third, the social structures of these OICs are characterized by a high level of diversity in terms of user backgrounds, expertise, and interests. This diversity brings a wealth of knowledge and perspectives to the community, which enhances the creativity and innovation potential of the community. Moreover, community managers actively seek to facilitate interactions and collaborations between users with complementary expertise and interests, which further enhances the value co-destruction process within the community.

The study of four open innovation communities (OICs) in business analytics reveals that these communities have several key characteristics in terms of their social structures. The implications of revealed dependencies for these social groups are particularly significant, as these communities heavily rely on the contributions and collaborations of their members to generate value co-creation. The high user engagement and participation, openness, and transparency foster a culture of trust and collaboration within the community, enhancing the value co-destruction process. The high level of diversity in terms of user backgrounds, expertise, and interests brings a wealth of knowledge and perspectives to the community, further enhancing the value co-destruction process. By understanding the dependencies that exist between members and the factors that influence their interactions, practitioners and policymakers can develop strategies and interventions to promote successful knowledge sharing and innovation, as well as policies and regulations that support the growth and sustainability of open innovation communities. This study’s findings can serve as a typical case study for academic research and provide valuable suggestions for the development of other OICs.

The empirical data used to test the hypotheses in this study were collected through an electronic questionnaire developed by Survey Monkey, a web-based application. The questionnaire’s link was shared on the selected communities’ platforms, and participation was voluntary without any financial incentives. To avoid duplication, respondents’ IP addresses were locked after completing the survey, and only one copy of the questionnaire was retained for each address. Out of the 1200 questionnaires sent to respondents, 366 were completed, yielding a valid response rate of 31%, which was deemed adequate for regression analysis in this study. The questionnaire was pre-tested before the official survey, and some questions were adjusted based on the test results.

This study employed rigorous measures to ensure the reliability of data sources. Specifically, samples lacking experiences of value co-destruction were excluded to ensure data accuracy. Participants were first introduced to the definition of value co-destruction and were asked to indicate whether they had experienced it before. Only those who met the criteria were allowed to complete the formal questionnaire, and were instructed to refer to that experience when answering subsequent questions. This process resulted in the removal of 15 questionnaires. Additionally, 30 questionnaires were excluded due to incomplete or missing data, resulting in a total of 321 valid questionnaires being obtained. To further examine the potential for nonresponse bias, this study divided the valid sample into two groups based on the order of receipt and conducted an independent sample t-test to assess whether significant differences existed in demographic characteristics, such as gender, age, education, and duration of community membership. The results of this analysis showed that t-values were not significant at the 0.05 level, indicating the absence of nonresponse bias in the study data. The basic characteristics of the valid sample, including gender, age, education, and duration of community membership, are presented in Table 1.

### 3.2. Measures Development

The process of developing a valid questionnaire requires a comprehensive and rigorous approach to ensure that the items selected accurately measure the intended constructs. To attain content validity, the present study utilized established measures as the basis for the questionnaire items, but also made necessary adaptations to suit the specific context and research objective. This was executed during the instrument survey construction, where three subject matter experts from the department of Management Information Systems were consulted to validate the accuracy and relevance of the selected items. Self-interest expectancy disconfirmation was measured using a scale developed by Nam et al. [43]. Social interaction expectancy disconfirmation was measured using a scale developed by Hsu et al. [59]. Self-worth expectancy disconfirmation was measured using a scale developed by Järvi et al. [60]. Psychological contract breach was measured using a scale developed by Marikyan et al. [39], which included transactional, relational, and ideological psychological contract breach. Value co-destruction was measured using a scale developed by Nadeem et al. [42]. Finally, perceived organizational status was measured using a scale developed by Ukeje et al. [48]. The study variables, along with the corresponding scale items used in the questionnaire, are detailed in Table 2.

Before the final version of the questionnaire was distributed among participants in OICs, it underwent a pilot testing phase to evaluate its clarity, comprehensibility, and ease of use. This phase involved the administration of the questionnaire to a small group of respondents who provided feedback on the wording, format, and overall structure of the questionnaire. The pilot testing process was critical in identifying any potential sources of confusion or ambiguity in the questionnaire, thereby providing an opportunity to refine and improve the items. To ensure a standardized response format among the participants, a Likert 7-point scale was employed for all measured variables. The participants were asked to provide subjective evaluations ranging from 1 (strongly disagree) to 7 (strongly agree) for each item. This utilization of the Likert scale allowed for a standardized response format, which reduced the potential for misinterpretation or bias in the responses. Finally, the questionnaire underwent a comprehensive review by multiple researchers and experts to evaluate its content validity. This review involved an examination of the questionnaire items in the context of the research question, construct definitions, and research model. The aim of this review was to ensure that the items were congruent with the intended constructs and accurately captured the data necessary to address the research objective of this study.

## 4. Data Analysis and Results

### 4.1. Testing for Common Method Variance

In this study, a single instrument was employed to collect data, which raises the possibility of common method variance as a potential concern. In order to investigate the presence of common method variance (CMV), the study utilized Harman’s single-factor test and marker variable tests, as recommended by Podsakoff et al. [61]. An exploratory factor analysis was conducted to assess whether a single underlying factor could account for the variance of all the measured variables [62]. The findings indicated that multiple factors with eigenvalues greater than 1.0 explained 79.5% of the total variance, with the first factor accounting for only 41.6% of the variance in the data, which is below the 50% threshold suggested by Podsakoff et al. [61]. Therefore, the results suggest that common method variance is not a significant issue in this study.

The marker variable technique [63] was also applied to further test for CMV by assessing the correlation between the marker variable (gender) and the theoretically unrelated variable (value co-destruction). The analysis yielded an estimate of 0.008 for the amount of method variance parceled out from other correlations, and the results indicated no significant difference between the original and adjusted correlation estimates. Given the results of the Harman’s one-factor test and the marker variable test, it can be inferred that the CMV in the collected data is not substantial. This finding suggests that the presence of common method bias in the data is not severe, and the measured variables are sufficiently distinct and not subject to significant biases stemming from the data collection method. As such, the study results can be attributed to the differences in the underlying constructs being measured, rather than the data collection method.

### 4.2. Testing for Validity and Reliability of Measurement Instrument

This study utilized several methods to assess the validity and reliability of a questionnaire’s measurement items, as recommended by Hair et al. [64]. Firstly, to ensure content validity, the study utilized well-established and validated measures from previous studies to measure all variables in the current study (refer to Table 2). Secondly, Cronbach’s alpha was employed to measure internal consistency, which evaluates the degree to which items within a questionnaire are interrelated. This statistical measure calculates the extent to which each item in a questionnaire is correlated with every other item. Thirdly, construct validity was tested through average variance extracted (AVE) to determine whether the questionnaire’s items relate to the construct they are intended to measure. Additionally, composite reliability (CR) was employed as an indicator of construct reliability that measures the degree to which the questionnaire items consistently measure their corresponding constructs. Finally, convergent and divergent validity were assessed using correlation analysis to determine whether the questionnaire’s items are correlated with other measures of the same construct (convergent validity) or unrelated constructs (divergent validity). Table 3 presents the results of factor loading, Cronbach’s alpha, average variance extracted (AVE), and composite reliability (CR), which are all indicators of reliability and validity used in questionnaire testing.

To further assess the questionnaire’s structural validity, this study utilized AMOS 22.0 for confirmatory factor analysis (CFA). When compared to other models, the eight-factor model demonstrated a superior fit to the actual data. The data fit results indicate that χ^2^/df = 1.881 < 2, *p* < 0.05, RMSEA = 0.044 < 0.06, CFI = 0.931 > 0.90, GFI = 0.822 > 0.80, IFI = 0.914 > 0.90, TLI = 0.917 > 0.90. These indicators suggest that the data in this study fit well with the CFA model. The fit indices provide a comprehensive assessment of the degree to which the observed data align with the proposed theoretical model. The validated factor analysis results are presented in detail in Table 4.

### 4.3. Correlation Analysis

Table 5 displays the descriptive statistics and correlation coefficients among the variables of this study. The means, standard deviations, and correlations are presented. The preliminary confirmation of the research hypotheses can be inferred from the significant correlations among the main research variables.

### 4.4. Hypotheses Testing

The present study utilized multiple regression analysis to test the research hypotheses. The decision to use multiple regression analysis was based on several justifications. First, multiple regression is a well-established and widely used method that enables the exploration of the relationships among multiple independent variables and a single dependent variable. Furthermore, multiple regression permits the testing of hypotheses and the identification of significant predictors, which is crucial in advancing theoretical understanding. In addition, Structural Equation Modeling (SEM) necessitates a larger sample size and more complicated data requirements, which were not feasible for the present research. Finally, the primary objective of the investigation was to examine the associations among a limited number of variables, and therefore, the use of SEM was deemed unnecessary.

The results of the regression analysis are presented in Table 6, Table 7 and Table 8, and the maximum value of the variance inflation factor (VIF) for each model variable was observed to be significantly lower than the critical value of 10. This indicates the absence of a significant multicollinearity problem.

In support of hypothesis H1a, the direct effect regression results of Model 4 in Table 6 reveal that self-interest expectancy disconfirmation has a significant positive effect on value co-destruction (β = 0.238, *p* < 0.001). Similarly, the mediating effect regression results of Model 5 in Table 6 demonstrate that the transactional psychological contract breach fully mediates the relationship between self-interest expectancy disconfirmation and value co-destruction (β = 0.476, *p* < 0.001), thus providing further support for hypothesis H2a.

Further, the direct effect regression results of Model 4 in Table 7 show that social interaction expectancy disconfirmation has a significant positive effect on value co-destruction, supporting hypothesis H1b (β = 0.335, *p* < 0.001). Similarly, the regression results of the mediating effect of Model 5 in Table 7 show that the relational psychological contract breach fully mediates between social interaction expectancy disconfirmation and value co-destruction (β = 0.586, *p* < 0.001), thereby supporting hypothesis H2b.

Moreover, the direct effect regression results in Table 8 reveal that self-worth expectancy disconfirmation has a significant positive effect on value co-destruction, supporting hypothesis H1c (β = 0.474, *p* < 0.001). Similarly, the regression results of the mediating effect in Table 8 demonstrate that the ideological psychological contract breach fully mediates the relationship between self-worth expectancy disconfirmation and value co-destruction (β = 0.711, *p* < 0.001), thereby providing further support for hypothesis H2c.

### 4.5. Testing for Moderating Effect

In this study, the interaction term [65] was used to examine the moderating role of perceived organizational status on the relationships between expectancy disconfirmation and psychological contract breach. By including interaction terms in regression models, researchers can gain a more nuanced understanding of the complex relationships between variables and identify potential moderating factors that may influence the strength or direction of these relationships. The results presented in Table 9 indicate that perceived organizational status does not significantly moderate the relationship between self-interest expectancy disconfirmation and transactional psychological contract breach or the relationship between social interaction expectancy disconfirmation and relational psychological contract breach. As a result, hypotheses H3a and H3b were not supported. Nevertheless, the findings reveal that perceived organizational status has a significant, negative moderating effect on the association between self-worth expectancy disconfirmation and ideological psychological contract breach (β = −0.086, *p* < 0.01), providing support for hypothesis H3c.

**Figure 2:**
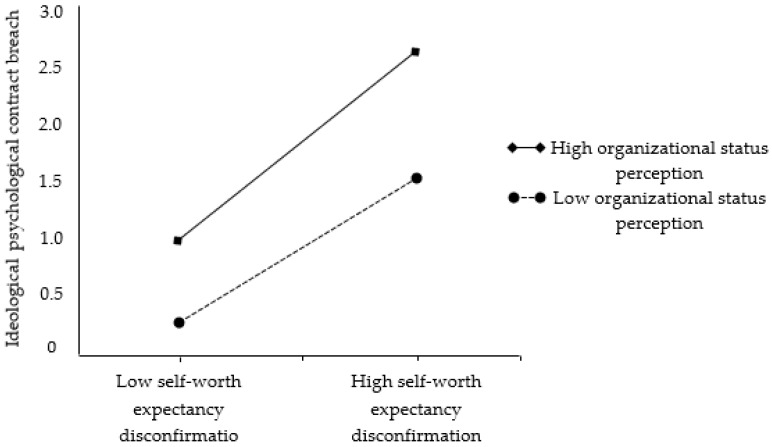
Moderating effect of perceived organizational status.

In order to gain a more comprehensive understanding of the data, this study produced interactive effect diagrams, as presented in Figure 2. The diagrams illustrate that when organizational status perception is low, there is a strong positive correlation between self-worth expectancy disconfirmation and ideological psychological contract breach. In contrast, when organizational status perception is high, this positive correlation weakens, which provides further support for Hypothesis 3c.

In summary, the results of this study, presented in Table 10, show that all three hypotheses (H1a–H1c) proposing a positive correlation between expectancy disconfirmation and value co-destruction were supported; highlighting the importance of aligning employees’ expectations with actual rewards and experiences. Additionally, all three hypotheses (H2a–H2c) suggesting that psychological contract breaches mediate the relationship between expectancy disconfirmation and value co-destruction were supported; underlining the role of psychological contracts in shaping users’ perceptions of value co-destruction in OICs. However, the hypotheses (H3a and H3b) proposing that perceived organizational status moderates the relationship between expectancy disconfirmation and psychological contract breaches were not supported. Only the hypothesis (H3c) suggesting that perceived organizational status moderates the relationship between self-worth expectancy disconfirmation and ideological psychological contract breach was supported. These findings underscore the need for organizations to pay close attention to managing employee expectations and psychological contracts to prevent value co-destruction in OICs.

## 5. Discussion

Open innovation communities (OICs) have become a significant source of external innovation for firms, and understanding the factors that contribute to value co-destruction in these communities is crucial for effective innovation management. This study aims to explore the relationship between expectancy disconfirmation and psychological contract breaches in OICs and examine the moderating role of perceived organizational status on this relationship. This section summarizes the key findings of the study and provides a critical discussion of their implications for theory and practice.

The findings of this study indicate that expectancy disconfirmation is a crucial factor that contributes to value co-destruction among community users in OICs. The results are consistent with the suggestion of Li et al. [35] that users have specific expectations when they join online communities and respond to other users’ expectations. Additionally, the results identify a higher-level expectancy beyond recognized benefits and relationships in OICs. This expectation centers on contributing to the community’s vision and noble mission, which aligns with users’ own self-interest expectancy. This finding provides a compelling rationale for users’ motivated participation in corporate mission-building activities [36] and even for behaviors that do not require remuneration [38]. The findings of this study also support Lv et al. [37] proposition that expectancy disconfirmation can result in value co-destruction within the context of open innovation. However, this study illuminates the process by which this occurs. It demonstrates that when community users experience disconfirmation in their self-interest, social interactions, and self-worth expectancies, they view their participation in innovation activities as “futile,” “lonely,” and “mediocre.” This perception makes them unwilling to contribute knowledge to community innovation activities, which ultimately results in the loss of a significant external source of innovation for the firm.

The findings of this study also reveal the important mediating role of psychological contract breach in the relationship between expectancy disconfirmation and value co-destruction in OICs. This finding provides empirical support for the open innovation ecosystem model proposed in previous studies [39,40,41] and underscores the significance of users as external innovation sources for virtual digital communities [42]. This study highlights the importance of the psychological contract between community users and enterprises, which is based on the belief that the community user will reciprocate the community’s contribution and involves the user’s belief in their own contributions and reciprocal rewards from the community. The findings of this study align with Blau’s notion [43] of the ideological dimension in the minds of OIC users, where users and community enterprises share a valuable idea and work towards it. This study validates prior research indicating that virtual communities prioritize collective goals over individual users [13,21] and sheds light on the potential consequences and mechanisms of this phenomenon. This study shows how disconfirmation in self-interest, social interaction, and self-worth expectancies can lead to psychological contract breach and discouraging users from participating in innovative activities within the community. As such, the findings from this study suggest that community managers should carefully consider individual users’ expectations and address their disconfirmation to maintain their psychological contracts and prevent value co-destruction.

The present study illuminates the moderating effect of perceived organizational status on the relationship between self-worth expectancy disconfirmation and ideological psychological contract breach in OICs. This finding is in line with prior research [24,66,67], which indicates that perceived organizational status influences attitudes and behaviors of traditional organizational employees. Specifically, this study suggests that individuals with a high perceived organizational status are more likely to view themselves as significant community members who have a duty to steer the community towards its goals and objectives. Furthermore, this study supports the notion that unequal status among interacting parties can result in value destruction [28,68,69]. These results provide a fresh perspective for understanding users’ civic conduct in OICs. The findings of this study are consistent with the work of Gander [47] and Hyun et al. [70], which proposes that perceived organizational status affects employee contribution and behavior. Additionally, Kaur et al. [33] have argued that unequal status can lead to value destruction. Building on these prior findings, this study demonstrates that perceived organizational status moderates the relationship between self-worth expectancy disconfirmation and ideological psychological contract breach in OICs. High-status users are better equipped to mitigate undesirable conditions in the community by engaging in equitable communication with community enterprises, aligning innovative activities with the community’s mission, and monitoring the provision of opportunities and spaces that meet users’ needs.

However, this study highlights that perceived organizational status does not have a moderating effect on the transactional and relational psychological contract breaches that arise from self-interest expectancy disconfirmation and social interaction expectancy disconfirmation. One plausible explanation for this finding is that users who experience such disconfirmations may not share the same values and objectives with the community as those who experience self-worth expectancy disconfirmation. As a result, they may be less invested in the community’s future and more likely to seek alternative options rather than actively engage with the community to address the breach in the psychological contract. This underscores the significance of having a long-term psychological contract, which has been linked to higher reliability, loyalty, trustworthiness, and lower mobility compared to a short-term, goal-oriented psychological contract. This finding aligns with Li et al. [31], who argue that employees with a long-term psychological contract dimension tend to exhibit higher reliability, loyalty, trust, and lower turnover compared to those with a short-term and goal-driven psychological contract dimension.

Despite perceived organizational status not moderating the impact of transactional and relational psychological contract breaches on user behavior in OICs, this study offers valuable insights into how psychological contract breaches shape users’ conduct and the factors that influence it. The current study supports the notion that the ideological psychological contract is a stronger bonding mechanism between OICs and users compared to transactional psychological contracts. Hence, the study suggests that the ideological psychological contract dimension is a more effective “adhesive” than other dimensions such as transactional psychological contract in connecting OICs to users.

## 6. Implications

### 6.1. Theoretical Implications

The present study presents several significant theoretical contributions to the existing literature on value co-destruction, enriching the field of open innovation as an innovation paradigm. By utilizing a large sample empirical research method, the value co-destruction research topic’s research methodology is expanded, enhancing the generalizability and applicability of the research conclusions. Furthermore, the study highlights the value co-destruction phenomenon in online open innovation communities, an area that has received little attention in previous research. This sheds light on the underlying mechanisms of value co-destruction between companies and users, contributing to the theoretical achievements of open innovation.

The theoretical implications of this study can be categorized into three key areas. Firstly, this study emphasizes the significance of incorporating a user-centered approach in open innovation communities. The findings reveal that community managers must prioritize creating a positive community culture that aligns with users’ expectations to ensure their continued participation and contributions towards community innovation activities. By utilizing the expectancy-disconfirmation theory and empirical evidence of psychological contract breach as a mediator in the relationship between expectancy disconfirmation and value co-destruction, this study provides a comprehensive perspective on the value co-destruction phenomenon. Thus, this study’s theoretical contributions are vital in advancing open innovation research and practices, facilitating the emergence of innovative products, services, and business models.

Secondly, the study’s organizational behavior perspective is a complementary addition to previous research that failed to consider the identity of users as external employees. Through the use of the expectancy-disconfirmation theory, the study introduces psychological contract breach and perceived organizational status as the mediating and moderating variables, providing a comprehensive perspective on the value co-destruction phenomenon. Empirical evidence is provided for the role of psychological contract breach as a mediator in the relationship between expectancy disconfirmation and value co-destruction, underscoring the potential consequences of ignoring individual users in achieving collective goals. The study emphasizes the potential consequences of ignoring individual users in achieving collective goals and offers insights into how community managers can maintain psychological contracts to prevent value co-destruction.

Finally, this study’s contributions highlight the significance of virtual digital communities in fostering open innovation. By focusing on the value co-destruction phenomenon in online open innovation communities, the study sheds light on an underexplored research area. The empirical evidence of psychological contract breach as a mediator in the relationship between expectancy disconfirmation and value co-destruction highlights the crucial role of users as external innovation sources for virtual digital communities. Therefore, the study’s contributions provide insights into how companies can leverage virtual digital communities to enhance their innovation capabilities. By taking into account the role of psychological contracts, companies can maintain strong relationships with users, foster a sense of ownership and responsibility among community members, and harness the potential of virtual digital communities as innovation ecosystems.

Overall, the findings of this study emphasize the importance of creating a positive community culture that aligns with users’ expectations to ensure their continued participation and contributions towards community innovation activities. The study provides empirical evidence for the role of psychological contract breach as a mediator in the relationship between expectancy disconfirmation and value co-destruction, underscoring the significance of the psychological contract between community users and enterprises. Additionally, the study offers insights into how community managers can maintain psychological contracts to prevent value co-destruction and highlights the crucial role of users as external innovation sources for virtual digital communities.

### 6.2. Managerial Implications

The present study offers valuable insights for managers seeking to foster positive interactions with users in open innovation communities (OICs) and navigate the complex terrain of community management. Specifically, the study sheds light on the phenomenon of value co-destruction, which refers to the negative consequences that can arise from user participation in OICs, and provides practical recommendations for community managers to prevent and mitigate these negative effects. In this section, we will elaborate and discuss in depth three of the key managerial implications of the study’s findings.

Firstly, the study highlights the importance of transparency and effective communication in all aspects of community interaction. Community managers should set clear rules and norms and describe rewards and incentives fairly to avoid cultural mismatches and ensure that users feel a sense of ownership and contribution to the enterprise mission. It is also important for community managers to actively listen to user feedback and incorporate it into activity selection and group formation processes, thereby promoting a collaborative and inclusive community culture. To effectively manage user expectations, community enterprises must utilize collaborative governance frameworks that prioritize transparency, accountability, and effective communication. By providing users with clear guidelines and expectations, community managers can prevent misunderstandings and foster productive partnerships between enterprises and users. This approach requires community managers to be proactive in their communication efforts, providing regular updates on community developments, and responding to user feedback in a timely and effective manner.

Secondly, the study highlights the importance of empowering users to manage the community and fostering a sense of responsibility and mission towards community development. By doing so, community managers can build a strong partnership with users and promote a positive and collaborative community culture. Community managers should avoid coming across as dominant or overly authoritative and instead work towards creating an environment where users feel valued and respected. Empowering users can take many forms, including providing opportunities for user-led activities and initiatives, inviting users to participate in decision-making processes, and offering leadership training programs. By involving users in these activities, community managers can create a sense of ownership and responsibility among community members, leading to increased engagement and productivity.

Finally, the study highlights the importance of effective communication and feedback mechanisms in preventing and resolving conflicts with users. In cases where users experience disconfirmations of their expectations, community managers should offer psychological counseling and chat sessions to appease them and resolve conflicts. In cases where users cannot be appeased, community managers should offer sincere apologies and compensation to avoid negative behaviors that could harm the community. Effective expectation management can serve as a potent tool for facilitating value co-creation between enterprises and users in OICs, ultimately determining the success or failure of enterprise-user collaborations. Community managers should work to foster shared norms and values among community members, create forums for constructive feedback and dialogue, and proactively address conflicts and issues that arise. By prioritizing effective conflict resolution and expectation management, community managers can ensure that value co-creation remains at the forefront of enterprise-user collaborations.

Overall, the study’s findings provide valuable guidance for community managers seeking to create a positive and collaborative environment that supports innovation and productive partnerships between enterprises and users. By prioritizing transparency, empowerment, and effective communication, community enterprises can foster a strong and engaged user community that drives innovation and growth. Effective expectation management and conflict resolution methods are also essential for successful enterprise-user collaborations, highlighting the importance of proactive and collaborative community management strategies.

## 7. Limitations and Prospects for Further Research

Although the present study provides valuable insights into the relationship between psychological contract breach and value co-destruction, it is not without limitations. One of the primary concerns is the use of cross-sectional data, which may limit the validity of the relationships between variables. Regrettably, due to resource constraints and other factors, the use of longitudinal data was not possible, which could have compromised the overall persuasiveness of the results. To address this limitation, future research should endeavor to utilize longitudinal data to validate the model and provide a more robust understanding of the relationships between variables.

Furthermore, there is ample room for further refinement of the model. Additional variables, such as trust, fairness, and justice, could be explored to gain a more comprehensive understanding of the relationship between psychological contract breach and value co-destruction. Additionally, expanding the chain from psychological contract breach to value co-destruction could offer deeper insights into the underlying mechanisms of this relationship. By scrutinizing the processes that drive value co-destruction, researchers could develop a more nuanced understanding of how psychological contract breach impacts organizational outcomes.

While the study verifies the existence of moderation effects, it does not delve into the underlying mechanisms of these effects in great detail. Specifically, future research could investigate the negative moderation effect of perceived status and how it operates to influence the relationship between psychological contract breach and value co-destruction. This would entail an in-depth analysis of the role of perceived status in shaping employee behavior and the psychological processes that underlie these effects. By addressing these limitations and expanding the scope of the model, future research has the potential to develop a more comprehensive understanding of the complex relationships between psychological contract breach and value co-destruction.

It is worth noting that while the single-factor test is a common approach to examine common method variance, it has its limitations. Future research could consider using other techniques, such as confirmatory factor analysis or multi-trait multi-method matrix, to further assess the potential impact of common method variance on the study’s findings. By addressing these limitations, future research can build on the present study and further our understanding of the dynamics between psychological contract breach and value co-destruction.

In addition to the limitations and prospects for further research outlined in the study, it is essential to note that the research was conducted in a specific context and industry, which may limit the generalizability of the findings to other settings. The study focused on open innovation communities in the technology industry, and it is unclear whether the same relationships between psychological contract breach and value co-destruction would hold in other industries or types of communities. Therefore, future research could aim to test the model and hypotheses in other contexts, such as other open innovation communities in different industries or other types of online communities.

Testing the model and hypotheses in other contexts would help to establish the generalizability of the findings and provide a more comprehensive understanding of the underlying mechanisms of value co-destruction. For instance, different types of communities may have unique characteristics that impact the relationships between psychological contract breach and value co-destruction. By testing the model in a variety of contexts, researchers could identify these nuances and develop a more nuanced understanding of how to prevent and mitigate value co-destruction in different settings.

Furthermore, examining the model in different industries could reveal unique challenges and opportunities for preventing value co-destruction. For example, the nature of the products or services offered by a company could influence user expectations and impact the likelihood of psychological contract breach. By testing the model in different industries, researchers could identify industry-specific factors that impact the relationships between psychological contract breach and value co-destruction and develop tailored interventions to prevent and mitigate the negative impacts of value co-destruction.

## 8. Conclusions

This study has explored the intricate link between psychological contract breach and value co-destruction in the context of open innovation communities. Through empirical analysis, the study found that psychological contract breach has a positive relationship with value co-destruction, which is moderated by perceived organizational status. The study identified self-interest expectancy disconfirmation as a predictor of transactional psychological contract breach, leading to value co-destruction. Additionally, social interaction expectancy disconfirmation predicts relational psychological contract breach, which in turn leads to value co-destruction. Self-worth expectancy disconfirmation predicts ideological psychological contract breach, which results in value co-destruction. The study also highlighted the moderating role of perceived organizational status in the ideological psychological contract breach resulting from self-worth expectancy disconfirmation.

The implications of these findings are significant for both researchers and practitioners in the field of open innovation communities. Firstly, the study contributes to the literature on psychological contract breach and value co-destruction by identifying the specific mechanisms underlying this relationship within the context of open innovation communities. Moreover, by highlighting the moderating role of perceived status, the study offers a more nuanced understanding of how psychological contract breach impacts employee behavior and organizational outcomes. For practitioners, the study suggests that organizations can take active steps to prevent value co-destruction resulting from psychological contract breach within open innovation communities. Specifically, it may be useful for community managers to prioritize transparency and clear communication in all aspects of community interaction, as well as empower users to manage the community and foster a sense of responsibility and mission towards community development. Additionally, organizations may consider implementing interventions to reduce psychological contract breaches and enhance the perceived status of employees, particularly those in lower-status positions.

In conclusion, the present study has provided valuable insights into the complex relationship between psychological contract breach and value co-destruction in the context open innovation communities. By addressing the issues identified in the study, organizations may be better equipped to prevent negative outcomes resulting from psychological contract breach and promote a more positive and collaborative work environment within open innovation communities.

## Figures and Tables

**Table 1:** Descriptive statistics of respondents (N = 321).

Sample Characteristics	Classification Criteria	Sample	
		Number	Percentage (%)
Gender	Male	227	70.7
	Female	94	22.01
Age	Less than 30 years old	147	45.8
	30~35 years old	112	34.9
	36~40 years	44	13.7
	Older than 40 years	16	5.0
Education level	College and below	34	10.6
	Bachelor’s degree	191	59.5
	Master’s degree	85	26.5
	PhD	11	3.4
Membership duration	<0.5 years	74	23.1
	0.5~1 year (excl.)	111	34.6
	1~2 years (excl.)	80	25.0
	2~3 years	56	17.4

**Table 2:** Study variables and corresponding scale items used in the questionnaire.

Variable	No.	Measurement Items	Source
Self-interest expectancy disconfirmation	1	The community did not recognize my efforts as much as I expected.	[43]
	2	I feel disappointed with the level of reward I received from the community.	
	3	I expected to receive more recognition from the community than I actually did.	
	4	The rewards I received from the community were not commensurate with my contributions.	
Social interaction expectancy disconfirmation	1	I expected to have more meaningful interactions with community members than I actually did.	[59]
	2	My contributions to the community did not lead to as many positive social relationships as I had expected.	
	3	The level of social support I received from community members was lower than I expected, given my contributions.	
	4	I feel disappointed with the quality of my relationships with community members, despite my active involvement in the community.	
Self-worth expectancy disconfirmation	1	My contributions to the community did not make me feel as valuable as I had expected.	[60]
	2	The level of recognition I received for my contributions to the community was lower than I expected, given their importance to the community’s mission.	
	3	I feel disappointed with the impact of my contributions to the community’s mission, given the effort I put in.	
	4	The community’s response to my attempts to persuade others to acknowledge the community’s mission was lower than I had expected.	
Transactional psychological contract breach	1	The community has not provided me with the rewards or recognition that I was promised for my contributions.	[39]
	2	I have put in more effort than the community has compensated me for.	
	3	The community has not fulfilled their obligations to provide timely and accurate feedback on my contributions.	
	4	The community has not provided me with the level of access or privileges that I was promised in exchange for my contributions.	
Relational psychological contract breach	1	The community has not provided me with the level of social support or recognition that I expected given my contributions.	
	2	I feel like the community has not upheld their end of the agreement in terms of building and maintaining a positive relationship with me.	
	3	The community has not provided me with opportunities to develop meaningful connections or relationships with other members.	
	4	I feel like the community has not been transparent or honest in their communication with me, which has damaged our relationship.	
Ideological psychological contract breach	1	I feel like the community’s values and mission have changed in a way that is inconsistent with my own beliefs and values.	
	2	The community’s actions or decisions have contradicted the values and mission they profess to uphold.	
	3	I feel like the community’s policies or practices are not consistent with the values and mission that they promote.	
	4	The community has not provided me with opportunities to engage in meaningful discussions or activities that align with my ideological beliefs.	
Value co-destruction	1	I feel that my contributions have not been valued or appreciated by the community, which has discouraged me from participating in future activities.	[42]
	2	The community’s decision-making processes have led to negative outcomes that have affected the quality of my contributions and decreased my willingness to participate in future activities.	
	3	The community’s interactions with me have been negative or unsupportive, which has decreased my motivation to contribute to future activities.	
	4	The community’s lack of responsiveness or engagement with my ideas or feedback has made me less interested in contributing to future activities.	
	5	The community’s culture or norms have created a hostile or unwelcoming environment that has made me reluctant to participate in future activities.	
Perceived organizational status	1	I believe that my contributions are highly valued by the community.	[48]
	2	I feel that my opinions and feedback are taken into account when the community makes decisions.	
	3	I perceive the community as being highly respected within the broader innovation community.	
	4	I believe that the community has a strong reputation for innovation and creativity.	

**Table 3:** Results of validity and reliability of the instrument and corresponding items.

Variable	No.	Factor Loading	Cronbach’s α	AVE	CR
Self-interest expectancy disconfirmation	1	0.807	0.820	0.627	0.826
	2	0.823			
	3	0.794			
	4	0.740			
Social interaction expectancy disconfirmation	1	0.738	0.832	0.651	0.902
	2	0.773			
	3	0.824			
	4	0.857			
Self-worth expectancy disconfirmation	1	0.816	0.870	0.673	0.828
	2	0.781			
	3	0.733			
	4	0.905			
Transactional psychological contract breach	1	0.913	0.792	0.682	0.855
	2	0.804			
	3	0.790			
	4	0.778			
Relational psychological contract breach	1	0.773	0.810	0.619	0.874
	2	0.804			
	3	0.837			
	4	0.906			
Ideological psychological contract breach	1	0.793	0.784	0.672	0.895
	2	0.752			
	3	0.903			
	4	0.913			
Value co-destruction	1	0.854	0.830	0.655	0.903
	2	0.770			
	3	0.762			
	4	0.773			
	5	0.824			
Perceived organizational status	1	0.827	0.812	0.702	0.915
	2	0.906			
	3	0.773			
	4	0.772			

**Table 4:** Results of the confirmatory factor analysis.

Model	χ^2^/df	CFI	GFI	IFI	TLI	RMSEA
Eight-factor model a	1.881	0.931	0.822	0.914	0.917	0.044
Six-factor model b	7.056	0.767	0.478	0.768	0.778	0.122
Four-factor model c	17.520	0.376	0.151	0.375	0.349	0.221

**Table 5:** Results of mean, standard deviation and correlation coefficient analysis for each variable.

Variable		Mean	StdDiv.	1	2	3	4	5	6	7	8	9	10	11
1	Gender	1.55	0.51											
2	Age	2.07	0.81	0.113 *										
3	Education level	2.24	0.75	−0.004	0.053									
4	Membership duration	2.38	1.04	0.038	0.074	0.852 *								
5	Self-interest expectancy disconfirmation	4.39	1.69	0.109 *	0.008	−0.066	−0.032							
6	Social interaction expectancy disconfirmation	4.45	1.68	0.045	0.026	0.005	−0.003	0.103						
7	Self-worth expectancy disconfirmation	4.51	1.69	−0.027	−0.117 *	−0.038	−0.094	0.109 *	0.106 *					
8	Transactional psychology contractual breach	4.29	1.71	0.086	0.006	−0.029	−0.015	0.667 *	−0.003	0.029				
9	Relational psychology contractual breach	4.28	1.58	0.039	0.042	−0.026	−0.067	0.001	0.696 **	−0.002	0.195 **			
10	Ideological psychology contractual breach	4.41	1.61	−0.029	−0.111 *	0.007	−0.031	0.017	0.059	0.699 **	0.212 **	0.243 **		
11	Value co-destruction	5.21	1.17	0.022	−0.072	−0.015	−0.084	0.231 **	0.336 **	0.485 **	0.420 **	0.540 **	0.698 *	
12	Perceived organizational status	3.97	1.60	0.002	−0.019	−0.069	−0.079	0.123 *	0.203 **	0.151 **	−0.389 **	−0.325 **	−0.352 **	−0.381 **

Note: ** *p* < 0.01, * *p* < 0.05; N = 321.

**Table 6:** Direct effect of self-interest expectancy disconfirmation and mediating effect of transactional psychological contract breach on value co-destruction.

Variable	Transactional Psychological Contract Breach			Value Co-Destruction	
	Model 1	Model 2	Model 3	Model 4	Model 5
Gender	0.086	0.015	0.041	0.016	0.009
Age	−0.005	−0.004	−0.069	−0.069	−0.067
Educational level	−0.050	0.037	0.208 *	0.238 *	0.221
Membership duration	0.026	−0.025	−0.257 *	−0.274 **	−0.263 **
Self-interest expectancy disconfirmation		0.667 ***		0.238 ***	−0.080
Transactional psychological contract breach					0.476 ***
$R^{2}$	0.009	0.445	0.025	0.080	0.205
$\Delta R^{2}$	0.004	0.437	0.014	0.067	0.192
F	0.752	57.738 ***	2.234	6.213 ***	15.451 ***
ΔF	0.752	283.325 ***	2.236	21.611 **	56.848 **
VIF	1.021	1.033	1.021	1.033	1.033

Note: *** *p* < 0.001, ** *p* < 0.01, * *p* < 0.05.

**Table 7:** Direct effect of social interaction expectancy disconfirmation and mediating effect of relational psychological contract breach on value co-destruction.

Variable	Relational Psychological Contract Breach			Value Co-Destruction	
	Model 1	Model 2	Model 3	Model 4	Model 5
Gender	0.041	0.011	0.041	0.026	0.020
Age	0.043	0.029	−0.069	−0.076	−0.093
Educational level	0.121	0.103	0.208 *	0.199 *	0.140
Membership duration	−0.174	−0.155	−0.257 *	−0.248 **	−0.158
Social interaction expectancy disconfirmation		0.694 ***		0.335 ***	−0.072
Relational psychological contract breach					0.586 ***
$R^{2}$	0.013	0.492	0.025	0.136	0.311
$\Delta R^{2}$	0.002	0.485	0.014	0.124	0.299
F	1.075	69.731 ***	2.236	11.338 ***	26.980 ***
ΔF	1.075	340.337 ***	2.236	46.622 ***	91.081 ***
VIF	1.021	1.023	1.021	1.023	1.023

Note: *** *p* < 0.001, ** *p* < 0.01, * *p* < 0.05.

**Table 8:** Direct effect of self-worth expectancy disconfirmation and mediating effect of ideological psychological contract breach on value co-destruction.

Variable	Ideological Psychological Contract Breach			Value Co-Destruction	
	Model 1	Model 2	Model 3	Model 4	Model 5
Gender	−0.011	−0.008	0.041	0.043	0.047
Age	−0.107	−0.032	−0.069	−0.018	0.006
Educational level	0.111	−0.012	0.208 *	0.141	0.133
Membership duration	−0.117	0.029	−0.257 *	−0.159	−0.179
Self-worth expectancy disconfirmation		0.698 ***		0.474 ***	−0.023
Ideological psychological contract breach					0.711 ***
$R^{2}$	0.017	0.491	0.025	0.243	0.498
$\Delta R^{2}$	0.006	0.484	0.014	0.233	0.492
F	1.495	69.471 ***	2.236	23.157 ***	26.044 ***
ΔF	1.495	335.863 ***	2.236	104.295 ***	185.478 ***
VIF	1.021	1.021	1.021	1.021	1.021

Note: *** *p* < 0.001, * *p* < 0.05.

**Table 9:** Moderating effect of perceived organizational status.

Variable	Model 1	Model 2	Model 3	Model 4	Model 5	Model 6	Model 7	Model 8	Model 9
Gender	0.015	0.012	0.015	0.011	0.009	0.008	−0.008	−0.005	−0.012
Age	−0.004	−0.010	−0.007	0.029	0.021	0.019	−0.032	−0.031	−0.027
Educational level	0.037	0.043	0.043	0.103	0.099	0.097	0.012	0.002	0.003
Membership duration	−0.025	−0.066	−0.065	−0.155 *	−0.189 ***	−0.187 ***	0.029	0.008	0.010
Self-interest expectancy disconfirmation	0.667 ***	0.725 ***	0.724 ***						
Social interaction expectancy disconfirmation				0.694 ***	0.793 ***	0.792 ***			
Self-worth expectancy disconfirmation							0.698 ***	0.766 ***	0.762 ***
Transactional psychological contract breach									
Relational psychological contract breach									
Ideological psychological contract breach									
Perceived organizational status		−0.480 ***	−0.480 ***		−0.493 ***	−0.492 ***		−0.466 ***	−0.469 ***
Self-interest expectancy disconfirmation × Perceived organizational status			−0.040						
Social interaction expectancy disconfirmation × Perceived organizational status						−0.046			
Self-worth expectancy disconfirmation × Perceived organizational status									−0.086 **
$R^{2}$	0.445	0.669	0.670	0.492	0.722	0.719	0.491	0.701	0.709
$\Delta R^{2}$	0.437	0.663	0.664	0.485	0.718	0.719	0.484	0.696	0.703
F	57.736	121.052	104.119	69.729	155.753	134.490	69.469	140.615	124.415
ΔF	57.736	243.916	1.642	69.729	298.931	2.646	335.861	253.788	8.854

Note: *** *p* < 0.001, ** *p* < 0.01, * *p* < 0.05.

**Table 10:** Results of path coefficients and hypotheses testing.

Hypothesis	Correlation	Test Results
H1a	There exists a positive correlation between self-interest expectancy disconfirmation and value co-destruction. The greater the discrepancy between self-interest expectations and the actual rewards received, the higher the likelihood of value co-destruction.	Supported
H1b	There exists a positive correlation between social interaction expectancy disconfirmation and value co-destruction. The greater the discrepancy between social interaction expectations and the actual experience of the activities, the higher the likelihood of value co-destruction.	Supported
H1c	There exists a positive correlation between self-worth expectancy disconfirmation and value co-destruction. The greater the discrepancy between self-worth expectations and the actual experience of the activities, the higher the likelihood of value co-destruction.	Supported
H2a	Transactional psychological contract breach mediates the relationship between self-interest expectancy disconfirmation and value co-destruction.	Supported
H2b	Relational psychological contract breach mediates the relationship between social interaction expectancy disconfirmation and value co-destruction.	Supported
H2c	Ideological psychological contract breach mediates the relationship between self-worth expectancy disconfirmation and value co-destruction.	Supported
H3a	Perceived organizational status moderates the relationship between self-interest expectancy disconfirmation and transactional psychological contract breach, with a weaker positive relationship between the two as perceived organizational status increases.	Not Supported
H3b	Perceived organizational status moderates the relationship between social interaction expectancy disconfirmation and relational psychological contract breaches, with a weaker positive relationship between the two as perceived organizational status increases.	Not Supported
H3c	Perceived organizational status moderates the relationship between self-worth expectancy disconfirmation and ideological psychological contract breach, with a weaker positive relationship between the two as perceived organizational status increases.	Supported

## Data Availability

Not applicable.

## References

[1] Daradkeh M. (2022). The Relationship Between Persuasion Cues and Idea Adoption in Virtual Crowdsourcing Communities: Evidence From a Business Analytics Community. Int. J. Knowl. Manag. IJKM.

[2] Daradkeh M. (2022). Innovation in Business Intelligence Systems: The Relationship Between Innovation Crowdsourcing Mechanisms and Innovation Performance. Int. J. Inf. Syst. Serv. Sect. IJISSS.

[3] Daradkeh M. (2022). A User Segmentation Method in Heterogeneous Open Innovation Communities Based on Multilayer Information Fusion and Attention Mechanisms. J. Open Innov. Technol. Mark. Complex..

[4] Daradkeh M. (2022). Lurkers versus Contributors: An Empirical Investigation of Knowledge Contribution Behavior in Open Innovation Communities. J. Open Innov. Technol. Mark. Complex..

[5] Daradkeh M. (2023). The Nexus between Business Analytics Capabilities and Knowledge Orientation in Driving Business Model Innovation: The Moderating Role of Industry Type. Informatics.

[6] Daradkeh M. (2021). The Influence of Sentiment Orientation in Open Innovation Communities: Empirical Evidence from a Business Analytics Community. J. Inf. Knowl. Manag..

[7] AbdelAziz K., Md Saad N., Thurasamy R. (2023). Analysing the factors influencing customer engagement and value co-creation during COVID-19 pandemic: The case of online modest fashion SMEs in Egypt. J. Islam. Mark..

[8] Arici H., Köseoglu M., Sökmen A. (2022). The intellectual structure of customer experience research in service scholarship: A bibliometric analysis. Serv. Ind. J..

[9] Bidar R., Jabbari M., Luck E. (2022). Value co-destruction in online collaborative networks. Eur. Manag. J..

[10] Ashraf M., Ahmad J., Hamyon A., Sheikh M., Sharif W. (2020). Effects of post-adoption beliefs on customers’ online product recommendation continuous usage: An extended expectation-confirmation model. Cogent Bus. Manag..

[11] Chang H., Zhang L. (2019). Psychological Contract Breach and Customer Satisfaction: A Study of Online Shopping. Serv. Mark. Q..

[12] Baron S., Warnaby G., Hunter-Jones P. (2014). Service(s) Marketing Research: Developments and Directions. Int. J. Manag. Rev..

[13] Cao Y., Lee J., Waung M. (2023). Cultivating organizational attraction: A resource view on psychological contracts of career development among interns. Pers. Rev..

[14] Castillo D., Canhoto A., Said E. (2021). The dark side of AI-powered service interactions: Exploring the process of co-destruction from the customer perspective. Serv. Ind. J..

[15] Chatterjee S., Rana N., Dwivedi Y. (2022). Assessing Consumers’ Co-production and Future Participation On Value Co-creation and Business Benefit: An F-P-C-B Model Perspective. Inf. Syst. Front..

[16] Venkatesh V., Goyal S. (2010). Expectation Disconfirmation and Technology Adoption: Polynomial Modeling and Response Surface Analysis. MIS Q..

[17] Rousseau D. (1998). The ‘Problem’ of the Psychological Contract Considered. J. Organ. Behav..

[18] Seeck H., Parzefall M. (2008). Employee agency: Challenges and opportunities for psychological contract theory. Pers. Rev..

[19] Plé L., Chumpitaz Cáceres R. (2010). Not always co-creation: Introducing interactional co-destruction of value in service-dominant logic. J. Serv. Mark..

[20] Kirova V. (2021). Value co-creation and value co-destruction through interactive technology in tourism: The case of ‘La Cité du Vin’ wine museum, Bordeaux, France. Curr. Issues Tour..

[21] Chen X., Schuster L., Luck E. (2023). The well-being outcomes of multi-actor inter-organisational value co-creation and co-destruction within a service ecosystem. J. Serv. Mark..

[22] Engen M., Fransson M., Quist J., Skålén P. (2021). Continuing the development of the public service logic: A study of value co-destruction in public services. Public Manag. Rev..

[23] Chen Y., Chen R., Hou J., Hou M., Xie X. (2021). Research on users’ participation mechanisms in virtual tourism communities by Bayesian network. Knowl. -Based Syst..

[24] Cluley V., Parker S., Radnor Z. (2023). Editorial: Public value for all? Considering the parameters of public value co-creation. Public Money Manag..

[25] Codá R., Silva Farias J., Dias C. (2022). Interactive Value Formation and Lessons Learned from COVID-19: The Brazilian Case. J. Qual. Assur. Hosp. Tour..

[26] Dolan R., Seo Y., Kemper J. (2019). Complaining practices on social media in tourism: A value co-creation and co-destruction perspective. Tour. Manag..

[27] Echeverri P., Skålén P. (2021). Value co-destruction: Review and conceptualization of interactive value formation. Mark. Theory.

[28] Frau M., Frigau L., Cabiddu F., Mola F., Wang C.L. (2023). Value Co-creation or Value Co-destruction? the Role of Negative Emotions in Consumer-Firm Interaction in the Social Media Platform. The Palgrave Handbook of Interactive Marketing.

[29] Oliver R. (1980). A Cognitive Model of the Antecedents and Consequences of Satisfaction Decisions. J. Mark. Res..

[30] Jumaan I., Hashim N., Al-Ghazali B. (2020). The role of cognitive absorption in predicting mobile internet users’ continuance intention: An extension of the expectation-confirmation model. Technol. Soc..

[31] Li L., Frethey-Bentham C., Juric B., Brodie R. (2023). A Negative Actor Engagement Scale for Online Knowledge-Sharing Platforms. Australas. Mark. J..

[32] Kandampully J., Bilgihan A., Li D. (2022). Unifying technology and people: Revisiting service in a digitally transformed world. Serv. Ind. J..

[33] Kaur J., Lavuri R., Parida R., Singh S. (2023). Exploring the Impact of Gamification Elements in Brand Apps on the Purchase Intention of Consumers. J. Glob. Inf. Manag. JGIM.

[34] Koc E., Yazici Ayyildiz A. (2022). An overview of tourism and hospitality scales: Discussion and recommendations. J. Hosp. Tour. Insights.

[35] Li M., Tuunanen T. (2022). Information Technology–Supported value Co-Creation and Co-Destruction via social interaction and resource integration in service systems. J. Strateg. Inf. Syst..

[36] Liang H. (2023). Dual effects of regulatory focus on work-related consequences: The mediating roles of psychological contracts. Manag. Decis..

[37] Lv X., Zhang R., Li Q. (2021). Value co-destruction: The influence of failed interactions on members’ behaviors in online travel communities. Comput. Hum. Behav..

[38] Madanaguli A., Dhir A., Talwar S., Clauss T., Kraus S., Kaur P. (2023). Diving into the uncertainties of open innovation: A systematic review of risks to uncover pertinent typologies and unexplored horizons. Technovation.

[39] Marikyan D., Papagiannidis S., Alamanos E. (2020). Cognitive Dissonance in Technology Adoption: A Study of Smart Home Users. Inf. Syst. Front..

[40] Mazhar M., Hooi Ting D., Zaib Abbasi A., Nadeem M., Abbasi H. (2022). Gauging customers’ negative disconfirmation in online post-purchase behaviour: The moderating role of service recovery. Cogent Bus. Manag..

[41] Mingione M., Leoni L. (2020). Blurring B2C and B2B boundaries: Corporate brand value co-creation in B2B2C markets. J. Mark. Manag..

[42] Nadeem W., Juntunen M., Shirazi F., Hajli N. (2020). Consumers’ value co-creation in sharing economy: The role of social support, consumers’ ethical perceptions and relationship quality. Technol. Forecast. Soc. Chang..

[43] Nam K., Baker J., Ahmad N., Goo J. (2020). Dissatisfaction, Disconfirmation, and Distrust: An Empirical Examination of Value Co-Destruction through Negative Electronic Word-of-Mouth (eWOM). Inf. Syst. Front..

[44] Naqvi M., Jiang Y., Naqvi M. (2021). Generating customer engagement in electronic-brand communities: A stimulus–organism–response perspective. Asia Pac. J. Mark. Logist..

[45] Ojuri O., Mills G., Opoku A. (2023). Exploring social value and their enablers as business models for sustainable water supply projects. Built Environ. Proj. Asset Manag..

[46] Taylor T., Darcy S., Hoye R., Cuskelly G. (2006). Using Psychological Contract Theory to Explore Issues in Effective Volunteer Management. Eur. Sport Manag. Q..

[47] Gander M. (2023). The psychological contracts of university professional staff: Expectations, obligations and benefits. Perspect. Policy Pract. High. Educ..

[48] Ukeje U., Lasisi T., Eluwole K., Titov E., Ozturen A. (2021). Organizational level antecedents of value co-destruction in hospitality industry: An investigation of the moderating role of employee attribution. Curr. Issues Tour..

[49] Owusu Yeboah A., Kwarteng M., Novak P. (2023). Social media marketing, value creation and firm’s sustainability performance: A study among young consumers. Aslib J. Inf. Manag..

[50] Pathak B., Ashok M., Tan Y. (2020). Value co-destruction: Exploring the role of actors’ opportunism in the B2B context. Int. J. Inf. Manag..

[51] Re B., Magnani G. (2023). Value co-creation processes in the context of circular entrepreneurship: A quantitative study on born circular firms. J. Clean. Prod..

[52] Quach S., Thaichon P. (2017). From connoisseur luxury to mass luxury: Value co-creation and co-destruction in the online environment. J. Bus. Res..

[53] Ranjan K., Read S. (2019). Bringing the individual into the co-creation of value. J. Serv. Mark..

[54] Ribeiro T., Costa B., Ferreira M., Freire O. (2023). Value co-creation in tourism and hospitality: A systematic literature review. Eur. Manag. J..

[55] Van den Groenendaal S., Freese C., Poell R., Kooij D. (2023). Inclusive human resource management in freelancers’ employment relationships: The role of organizational needs and freelancers’ psychological contracts. Hum. Resour. Manag. J..

[56] van Klyton A., Tavera-Mesias J., Castaño-Muñoz W. (2022). Value co-creation and co-destruction in the first cashless society in Colombia—A middle range theory approach. Inf. Technol. People.

[57] Daradkeh M. (2021). Exploring the Usefulness of User-Generated Content for Business Intelligence in Innovation: Empirical Evidence From an Online Open Innovation Community. Int. J. Enterp. Inf. Syst. IJEIS.

[58] Daradkeh M. (2021). Analyzing Sentiments and Diffusion Characteristics of COVID-19 Vaccine Misinformation Topics in Social Media: A Data Analytics Framework. Int. J. Bus. Anal..

[59] Hsu P., Nguyen T., Huang J. (2021). Value co-creation and co-destruction in self-service technology: A customer’s perspective. Electron. Commer. Res. Appl..

[60] Järvi H., Kähkönen A., Torvinen H. (2018). When value co-creation fails: Reasons that lead to value co-destruction. Scand. J. Manag..

[61] Podsakoff P., MacKenzie S., Lee J., Podsakoff N. (2003). Common Method Biases in Behavioral Research: A Critical Review of the Literature and Recommended Remedies. J. Appl. Psychol..

[62] Malhotra N., Kim S., Patil A. (2006). Common Method Variance in IS Research: A Comparison of Alternative Approaches and a Reanalysis of Past Research. Manag. Sci..

[63] Daradkeh M. (2021). An Empirical Examination of the Relationship Between Data Storytelling Competency and Business Performance: The Mediating Role of Decision-Making Quality. J. Organ. End User Comput. JOEUC.

[64] Hair J., Hult G., Ringle C., Sarstedt M. (2017). A Primer on Partial Least Squares Structural Equation Modeling PLS-SEM.

[65] Baron R., Kenny D. (1986). The Moderator-Mediator Variable Distinction in Social Psychological Research: Conceptual, Strategic, and Statistical Considerations. J. Personal. Soc. Psychol..

[66] Das K., Patel J., Sharma A., Shukla Y. (2023). Creativity in marketing: Examining the intellectual structure using scientometric analysis and topic modeling. J. Bus. Res..

[67] De Ruiter M., Schalk R., Bergum S., Peters P., Vold T. (2023). The Employment Relationship Amidst and Beyond the COVID-19 Pandemic: The Role of (Responsible) Inclusive Leadership in Managing Psychological Contracts. Virtual Management and the New Normal New Perspectives on HRM and Leadership Since the COVID-19 Pandemic.

[68] Fazal-e-Hasan S., Ahmadi H., Sekhon H., Mortimer G., Sadiq M., Kharouf H., Abid M. (2023). The role of green innovation and hope in employee retention. Bus. Strategy Environ..

[69] Frau M., Cabiddu F., Frigau L., Tomczyk P., Mola F. (2023). How emotions impact the interactive value formation process during problematic social media interactions. J. Res. Interact. Mark..

[70] Hyun S., Kim J., Liu Y. (2023). Equal gains and pains? Analyzing corporate financial performance for industrial corporate social performance leaders and laggards. J. Bus. Res..

